# Lung physiological changes during awake prone positioning in non-intubated hypoxemia patients

**DOI:** 10.1186/s13054-023-04672-7

**Published:** 2023-10-13

**Authors:** Xiaolu Shao, Juqin Shao

**Affiliations:** 1 Department of Intensive Care, Zhejiang Hospital, 1229 Gudun Road, Hangzhou, 310013 Zhejiang People’s Republic of China; 2 Department of geriatrics, Zhejiang Hospital, 310013 Zhejiang Hangzhou, People’s Republic of China

Dear Editor,

In a recent randomized trial [1], Dr. Nay et al. investigated the efficacy of awake prone positioning versus usual care in hypoxemic COVID-19 patients in medical wards. A total of 268 patients were included. The authors reported that the composite primary outcome (non-invasive ventilation (NIV) or intubation or death within 28 days) was comparable between the two groups. However, in the predefined subgroup analysis, awake prone positioning was associated with a lower rate of the composite primary outcome in the subgroup with initial SPO_2_ ≥ 95%, compared to the usual care group, while it was associated with a higher rate of the composite primary outcome in the subgroup with initial SPO_2_ < 95% (p for interaction = 0.019).

As the author inferred that SPO_2_ of 95% at a flow of 5 L/min corresponds to a PaO_2_/FiO_2_ ratio of 200 mmHg, the result suggested that prone positioning showed clinical benefit only in patients with mild hypoxemia (PaO_2_/FiO_2_ > 200 mmHg). This finding is contrary to the current consensus on acute respiratory failure (ARF), which suggests that prone positioning ventilation reduced mortality only in ARF patients with severe hypoxemia (PaO_2_/FiO_2_ < 150 mmHg) [2].

We think that two things need to be clarified before applying these findings to clinical practice: (1) Why prone positioning showed no overall benefit in these hypoxemic COVID-19 patients. (2) Why prone positioning showed a beneficial trend in the subgroup with initial SPO_2_ ≥ 95%, which is conflicting with consensus in ARF [2].

First, according to the current evidence, the efficacy of prone positioning depends on the respiratory support during prone positioning [3–5]. In a meta-analysis [4] including 29 studies of COVID-19-related ARF, a low intubation rate was only seen in patients who received advanced respiratory support (such as high-flow nasal cannula (HFNC) or NIV) but not in those with conventional oxygen therapy. Evidence has proved that prone positioning can lead to changes in pulmonary re-inflation and ventilation–perfusion matching. However, these physiological changes may rely on advanced respiratory support in non-intubated patients. For instance, respiratory support through HFNC/NIV can increase the end-expiratory lung volume, reduce intrapulmonary shunt fraction, and improve regional tidal volume distribution. Under this condition, prone positioning can improve lung compliance and recruitment, promote more uniform lung inflation, enhance oxygenation, and prevent ventilator-induced lung injury [5, 6], finally improving prognosis. However, under conventional oxygen therapy, prone positioning alone may not be enough to alter the outcome. In addition, the duration of prone positioning also plays a crucial role in prone positioning therapy. A meta-analysis [6] including eight randomized trials in ARDS showed that the duration of prone positioning determined the decrease or not of mortality, as only prone positioning more than 12 h/d can improve the prognosis. However, in the current study, the median time spent each day in the prone positioning within 72 h of randomization was only 90 min. In addition, more than 95% of patients received conventional oxygen therapy at enrollment. Thus, prone positioning showed no benefit in the overall population.

Second, the current study showed that awake prone positioning improved outcome in patients with mild hypoxemia (PaO_2_/FiO_2_ ≥ 200 mmHg), which is different from the current consensus that prone positioning only exhibits benefit in severe ARF patients receiving mechanical ventilation. As described above, previous evidence suggests that lung physiology changes during prone positioning largely depend on advanced respiratory support [3, 4], such as invasive/non-invasive mechanical ventilation, HFNC, etc. However, in the current study, more than 95% of patients received conventional oxygen therapy. Thus, we infer that the benefits of the awake prone position in patients with mild hypoxemia (PaO_2_/FiO_2_ ≥ 200 mmHg) may not be through the typical physiological changes (such as improving ventilation–perfusion matching, increasing end-expiratory lung volume, more uniform distribution of tidal volume, and alterations in chest wall mechanics [6]), but rather through improved lung ventilation by promoting sputum excretion.

Finally, we thank Dr. Nay et al. for their outstanding job, and we suggest that more research is needed to explore the different mechanisms of prone ventilation in intubated and non-intubated patients with ARF.

## Data Availability

Not applicable.

## References

[1] Nay MA, Hindre R, Perrin C, Clement J, Plantier L, Seve A, Druelle S, Morrier M, Laine JB, Colombain L (2023). Prone position versus usual care in hypoxemic COVID-19 patients in medical wards: a randomised controlled trial. Crit Care.

[2] Bloomfield R, Noble DW, Sudlow A (2015). Prone position for acute respiratory failure in adults. Cochrane Database Syst Rev.

[3] Pavlov I, Li J, Kharat A, Luo J, Ibarra-Estrada M, Perez Y, McNicolas B, Poole D, Roca O, Vines D (2023). Awake prone positioning in acute hypoxaemic respiratory failure: an international expert guidance. J Crit Care.

[4] Li J, Luo J, Pavlov I, Perez Y, Tan W, Roca O, Tavernier E, Kharat A, McNicholas B, Ibarra-Estrada M (2022). Awake prone positioning for non-intubated patients with COVID-19-related acute hypoxaemic respiratory failure: a systematic review and meta-analysis. Lancet Respir Med.

[5] De Bels D, Redant S, Honore PM (2021). Prone positioning in coronavirus disease 2019 patients with acute respiratory distress syndrome: How and when is the best way to do it?. J Transl Int Med.

[6] Munshi L, Del Sorbo L, Adhikari NKJ, Hodgson CL, Wunsch H, Meade MO, Uleryk E, Mancebo J, Pesenti A, Ranieri VM (2017). Prone position for acute respiratory distress syndrome. A systematic review and meta-analysis. Ann Am Thorac Soc.

